# Supplementary material for: Acetone Sensing and Catalytic Conversion by Pd-Loaded SnO2
Source: Materials (Basel). 2021 Oct 9;14(20):5921. doi: 10.3390/ma14205921 (PMC8540906; doi:10.3390/ma14205921)
Supplement: Supplementary file 1 [file materials-14-05921-s001.zip › materials-1383995-supplementary.pdf]

Acetone Sensing and Catalytic Conversion by Pd-loaded SnO_2

Supporting Information

Pascal M. Gschwend[1], Florian M. Schenk[1], Alexander Gogos[2,3], Sotiris E. Pratsinis[1]*

[1] Particle Technology Laboratory, Institute of Energy and Process Engineering, Department of Mechanical and Process Engineering, ETH Zurich, Sonneggstrasse 3, CH-8092 Zurich, Switzerland

[2] Particles-Biology Interactions, Department of Materials Meet Life, Swiss Federal Laboratories for Materials Science and Technology (Empa), Lerchenfeldstrasse 5, CH-9014 St. Gallen, Switzerland

[3] Nanoparticle Systems Engineering Laboratory, Institute of Energy and Process Engineering, Department of Mechanical and Process Engineering, ETH Zurich, Sonneggstrasse 3, CH-8092 Zurich, Switzerland

* Corresponding author: pratsinis@ethz.ch

Experimental

Synthesis of Sensing Particles

Pure SnO_2 nanoparticles were prepared by flame spray pyrolysis as before [18]. In short, tin(II)-ethylhexanoate (STREM Chemicals, purity >99%) was diluted with xylene (Aldrich, purity >96%) to a final metal concentration of 0.5 M. The precursor solution was fed at 5 ml/min through a capillary tube and dispersed with oxygen (5 L/min) from the adjacent annulus into a spray of fine droplets, that is ignited and supported by a surrounding ring-shaped, premixed flamelet (CH_4/O_2 = 1.25/3.25 L/min). The spray flame was sheathed by 5 L/min of O_2 fed through the adjacent annulus to ensure complete precursor combustion. The powder was collected on a glass-fiber filter (Albet-Hahnemuehle, GF6, 257 mm diameter) with the aid of a vacuum pump (Busch, Seco SV 1025 C). The as-prepared powders were removed from the above filter with a spatula, sieved (0.25 mm stainless steel sieve) to remove glass-fiber residues and then annealed in air for five hours at 500 °C (Carbolite CWF 1300).

Palladium was then photo-deposited [19] onto pure flame-made and annealed SnO_2 at a concentration of 0, 0.1, 0.2, 0.5, 1 and 3 mol %. To this end, one gram of pure, annealed SnO_2 was dispersed in 100 ml of a water-ethanol mixture (4:6 volume ratio) in a glass beaker (diameter = 7 cm, height = 10 cm) using an ultrasonic bath for 10 min. In another beaker, an appropriate amount of Pd-nitrate solution ($Pd(NO_3)_2$, 10 wt% Pd, Alfa Aesar) was mixed with distilled water to a total volume of 100 ml, which was then added to the SnO_2 dispersion. After 2 min of magnetic stirring at 500 rpm, the beaker was illuminated for 4 h by a UV-lamp (302 nm, 8 W) placed on top of the beaker. Afterwards, the entire suspension was washed three times by centrifugation (10 min, 7500 RCF, Eppendorf centrifuge), the supernatants discarded and the pellet redispersed with distilled water under intermediate ultrasonication. Subsequently, the washed powder was dried in a vacuum oven (SalvisLab, vacucenter) for 12 h at 50 °C and 50 mbar, followed by grinding the collected powder in an agate mortar. Afterwards, the Pd-loaded (0 – 3 mol%) SnO_2 powders were annealed again at 500 °C for 5 h to bond the Pd to the SnO_2 support [20].

Powder characterization

The particle composition was investigated by X-ray diffraction (XRD, Bruker D2 Phaser) at 30 kV and 10 mA at 2θ (Cu K_α) of 15 – 70°, with a scanning speed of 0.3 s/step and a step size of 0.0203°. The patterns and crystal sizes were evaluated with the software TOPAS 4 (Bruker) and Rietveld fundamental parameter refinement with the reference files of cassiterite SnO_2 (PDF 41-1445), Pd (PDF 01-1310) and PdO (PDF 43-1024). The specific surface area (SSA) of powders was determined by five-point N_2 adsorption using the Brunauer-Emmett-Teller (BET) method at 77 K (Micromeritics,

TriStar 2 Plus). Prior to these measurements, powders were degassed under nitrogen for at least an hour at 150 °C. Particle sizes (d_{BET}) were calculated using the densities of SnO_2 (6.95 g/cm^3) and PdO (8.3 g/cm^3). The noble metal dispersion was determined on an Autochem II (Micromeritics) by CO-pulse chemisorption at 40 °C using a He flow of 50 ml/min and 5 % CO in He as loop gas at 50 ml/min. Prior to such measurements, samples were reduced using 5 % H_2 in Ar (50 ml/min) at 150 °C (heated at 10 °C/min with 30 min holding time at 150 °C). An adsorption stoichiometry (Fagherazzi, et al., 2000) of Pd:CO = 2 was assumed to calculate the metal dispersion and resulting Pd size (hemispherical particle shape).

For electron microscopy analysis, spatula tips of the dry material were mixed with 250 µl ethanol (>99.9% purity, LiChrosolv®, Merck) in a 1.5 ml eppendorf tube. The sample was then sonicated for 2 min and left to stand/sediment for 2 more min. Subsequently, 10 µl of the supernatant were drawn through a copper lacey carbon grid (EM resolutions, UK) using a tissue and the grid was washed three times with ultrapure water. Specimens were then imaged by TEM (transmission electron microscopy, Talos F200X, Super-X EDS, 4 detector configuration, FEI, USA) at an accelerating voltage of 200 kV. For acquisition of HAADF-STEM (high-angle annular dark-field scanning transmission electron microscopy) combined with EDX (energy-dispersive x-ray) element maps, a beam current of ≈8 nA was used. The data were processed using the software Velox 3.0.0.815 (Thermo fisher/FEI, USA). Size distribution of Pd-clusters have been extracted using the NanoDefine Particle Sizer plug-in for ImageJ.

For chemical analysis, the Pd-loaded particles were treated in a quartz reactor at 150°C for 30 min with a reducing atmosphere (5 % H_2 in Ar, 100 ml/min) at 150 °C) to reduce all PdO_x species to leachable Pd. Then, 80 mg of the reduced powder were added to 80 ml of 10% nitric acid (Fisher Scientific, 65% HNO_3 in water, purity 99.99%) in distilled water in a round-bottom flask (100 ml), heated in an oil-bath at 60 °C under magnetic stirring for 4h. The undissolved SnO_2 particles were separated by centrifugation (10 min, 7197 RCF, Eppendorf centrifuge 5430) from the leachate containing dissolved Pd. The solution was analyzed by ICP-OES (inductively coupled plasma optical emission spectrometry, Varian 720-ES axial) to determine the Pd loading. A Pd standard solution (Supelco Palladium standard, $Pd(NO_3)_2$ in HNO_3, 1000 mg/l, Pd certipur, MilliporeSigma) was used for calibration.

<u>Sensor Fabrication</u>

The Pd-containing SnO_2 particles were mixed with 1,2-propanediol (Aldrich, purity >99.5%) to form viscous and homogeneous pastes. Sensing films were prepared by doctor-blading [18] the pastes with a razor blade onto Al_2O_3 sensor substrates (15x13x0.8 mm, Electronic Design Centre Case

Western Reserve University, Electrode type #103). The substrates contained interdigitated Pt electrode pairs (sputtered, 350 µm width and spacing) and a Pt resistance temperature detector (RTD) on the front while featuring a Pt heater on the back. During doctor-blading, a 15 µm thick aluminum foil was used to cover the RTD and edges of the substrate. The sensors were dried in ambient air for 4 h and placed in an oven (Carbolite, CWF 1300) at 80 °C for further evaporation of the solvent. No further annealing was done before sensing testing following Korotcenkov et al. [21].

Sensor measurements

The sensors were tested as described previously [18]. In brief, 1 L/min of synthetic air was partially bubbled through distilled water to attain 50 % relative humidity (RH). Acetone was admixed from calibrated gas standards to achieve a 1 ppm concentration. The sensors were first heated to 350 °C by supplying a DC voltage through their heater. Subsequently, the sensor measurements were performed with decreasing temperature steps down to 150 °C. The sensor response (S) was defined as $S = \frac{R_{air}}{R_{analyte}} - 1$ wherein R_{air} and $R_{analyte}$ are the sensing film resistance in synthetic air (including RH) without and with analyte, respectively. The sensor response and recovery times were defined as the times needed to reach or recover 90 % of the resistance change during or after analyte exposure, respectively. The limit of detection was calculated at a signal-to-noise ratio of 3, where the noise was defined as one standard deviation of the film baseline resistance. The corresponding concentration was found by linearly extrapolating the sensor response at low concentrations through the origin.

Catalytic measurements

Catalytic measurements were done as described previously [22] with a small modification: The catalytic packed beds consisted of only 20 mg particles in a quartz glass tube with 4 mm inner diameter, that were fixed with quartz wool at both ends. The reactor was placed inside a furnace and 0.15 L/min of the same gas mixtures as for the sensing flowed through the tube. The off-gas was analyzed in real-time by a PTR-ToF-MS (Proton transfer reaction time of flight mass spectrometry, IONICONC, PTR-ToF-MS 1000, Austria) to its outlet. Acetone concentrations were investigated at mass-to charge (m/z) ratio (Müller, et al., 2014) of 59.045 and the gas conversion was calculated by $c = 1 - \frac{outlet\ concentration}{inlet\ concentration}$.

Supporting Figures

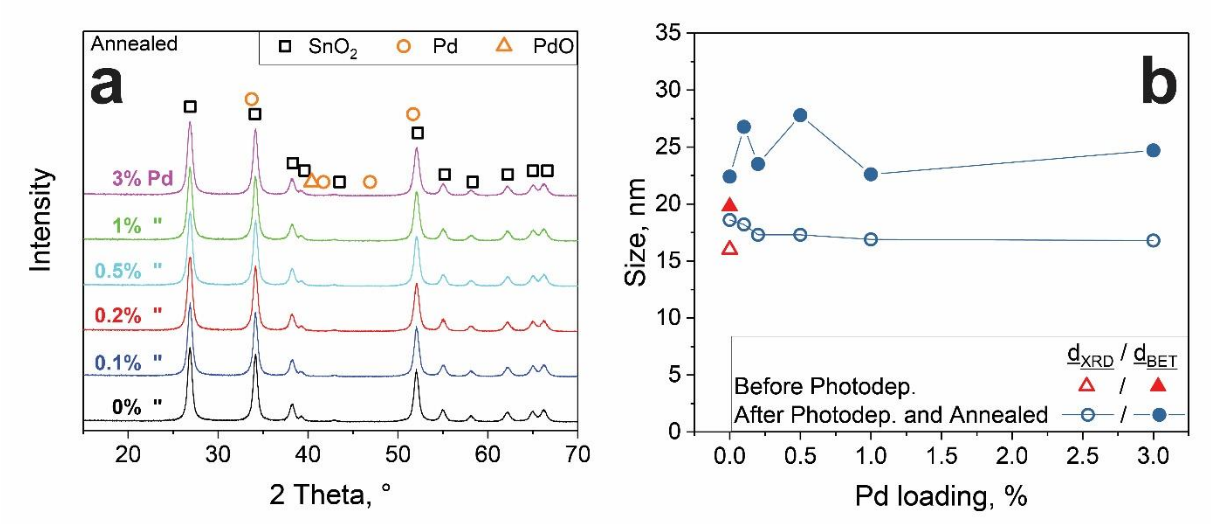

Figure S1: a) XRD patterns of SnO_2 with 0 – 3 mol% photodeposited palladium. No differences can be observed between them. **b)** Crystal (d_{XRD}, open symbols) and primary particle sizes (d_{BET}, filled symbols) before (triangles) and after photodeposition of Pd and annealing (circles).

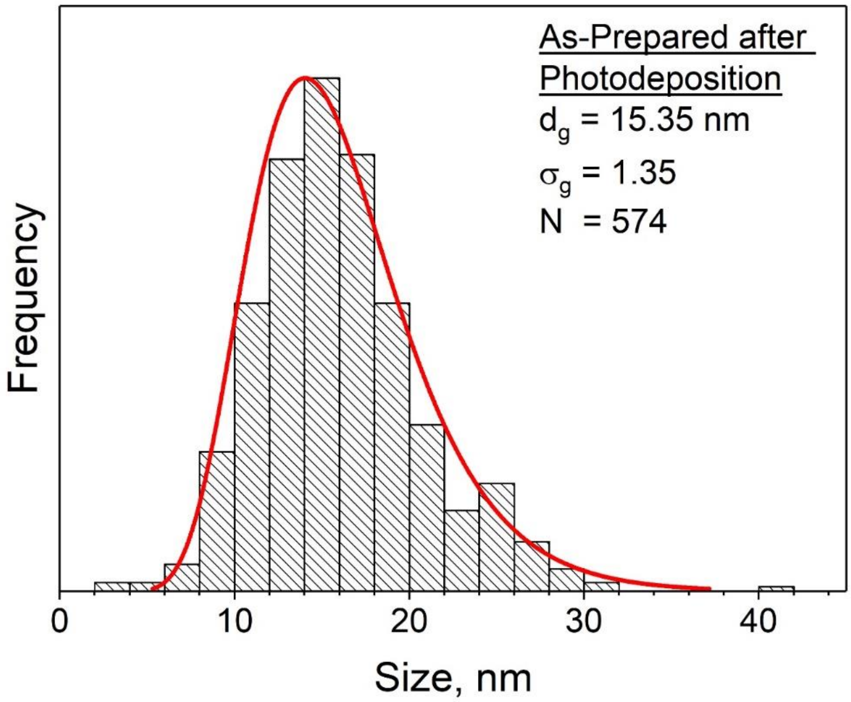

Figure S2: Primary particle size distribution from electron microscopy of SnO_2 particles (before the second annealing step) along with a fitted log-normal distribution (red line). The inset gives the geometric mean diameter (d_g) and standard deviation (σ_g) based on 574 counted particles (N), yielding almost identical results to the values after the second annealing step (Figure 1d). Therefore, the second annealing step had no effect on the SnO_2 size observed my microscopy.

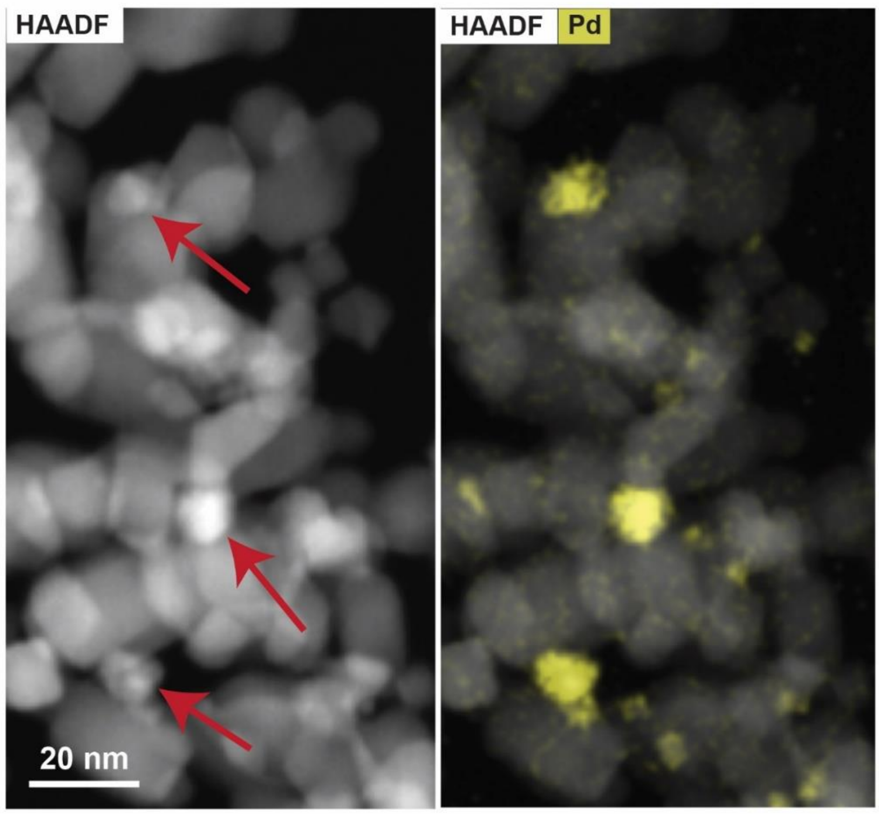

Figure S3: a) HAADF-STEM image of 3 mol% Pd-SnO_2 particles and **b)** an overlay with a Pd mapping (yellow). Due to the similarity in atomic number (thus Z-contrast), Pd particles cannot clearly be distinguished from SnO_2 (Examples highlighted with red arrows). Only the elemental mapping reveals the Pd clusters.

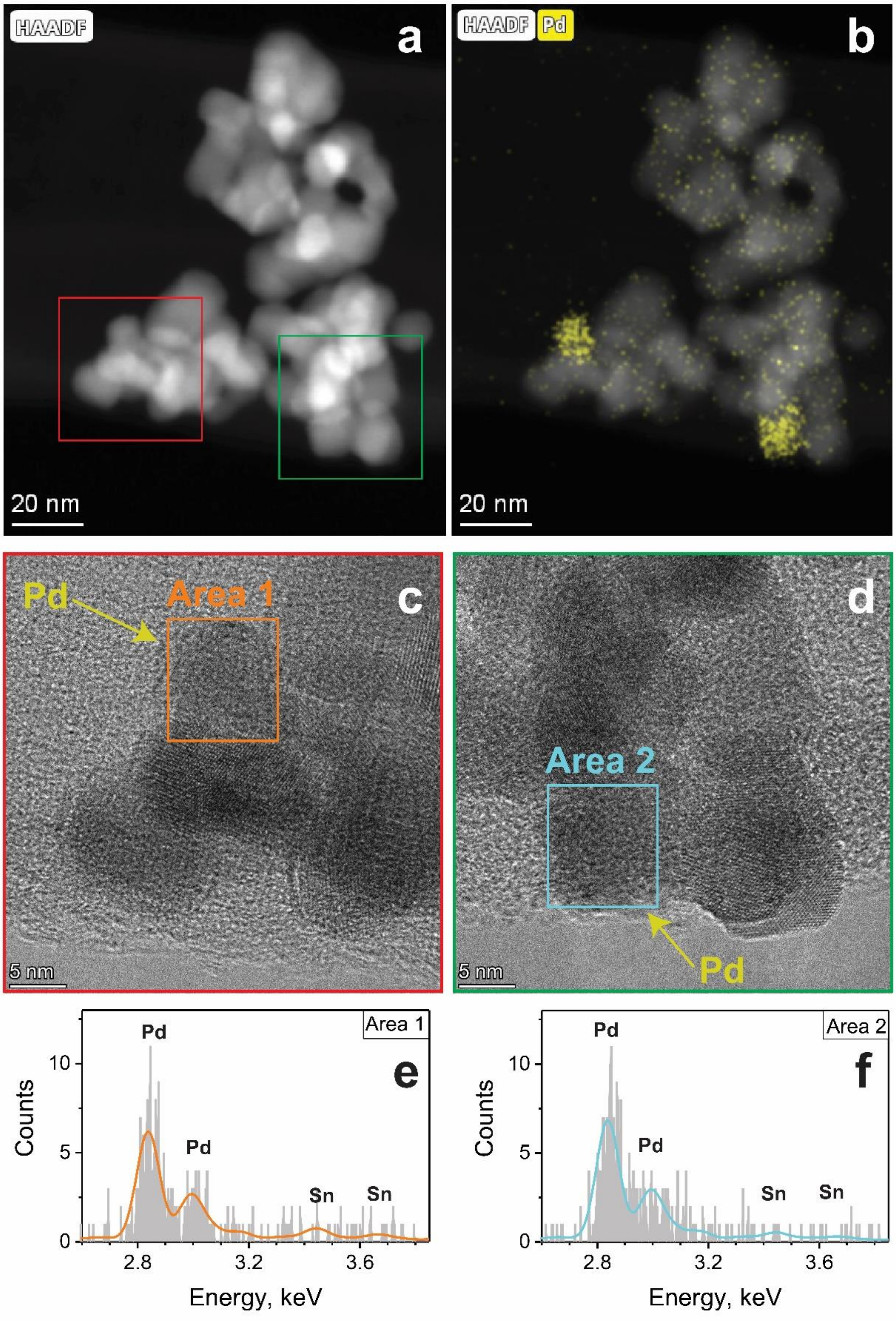

Figure S4: a) HAADF-STEM image of 3 mol% Pd-loaded SnO_2 (annealed) and **b)** combined with Pd elemental mapping. Two regions of interest have been identified with spots, where almost entirely Pd is present. These two regions have been further imaged with HR-TEM (**c** and **d**). No lattice fringes could be observed for the Pd-particles, while the neighboring SnO_2 particles clearly exhibited fringes, indicative of crystallinity. The EDX-spectra of the supposed PdO_x-particles (**e** and **f**, based on regions highlighted in Figures c and d) confirm the presence of mostly Pd. Therefore, the PdO_x particles are considered to be rather amorphous.

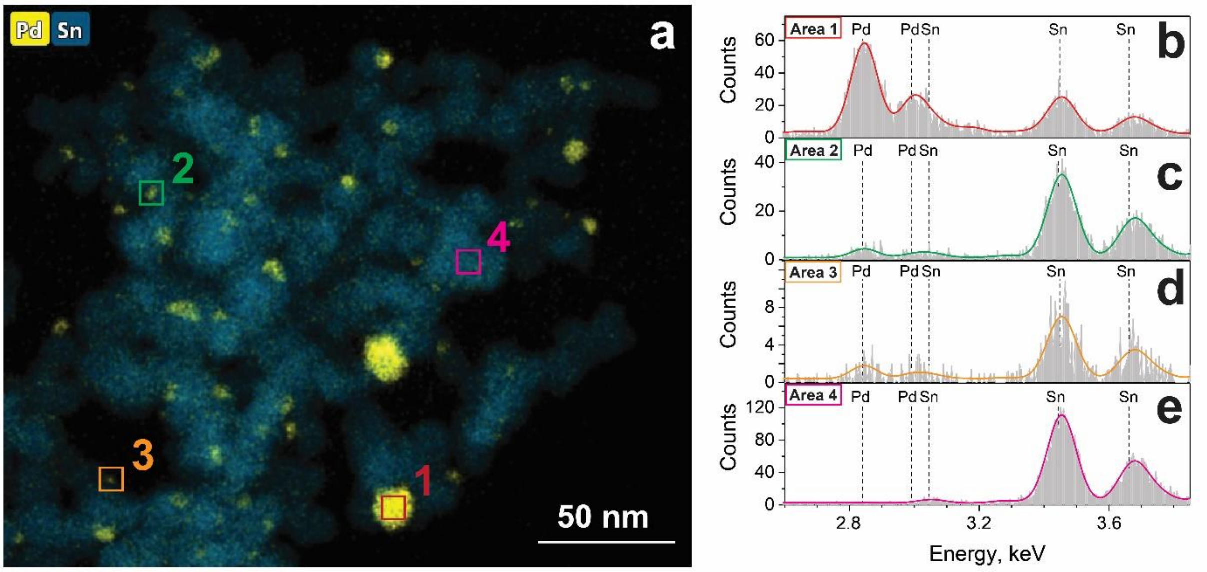

Figure S5: a) Elemental maps of Pd (yellow) and Sn (blue) of as-prepared 3 mol% Pd-loaded SnO_2. The EDX spectra of selected areas are depicted, specifically a large Pd cluster (Area 1, spectrum in **b**), a medium-sized cluster (Area 2, spectrum in **c**), a small Pd cluster (Area 3, spectrum in **d**), and a Pd-free SnO_2 (Area 4, spectrum in **e**).

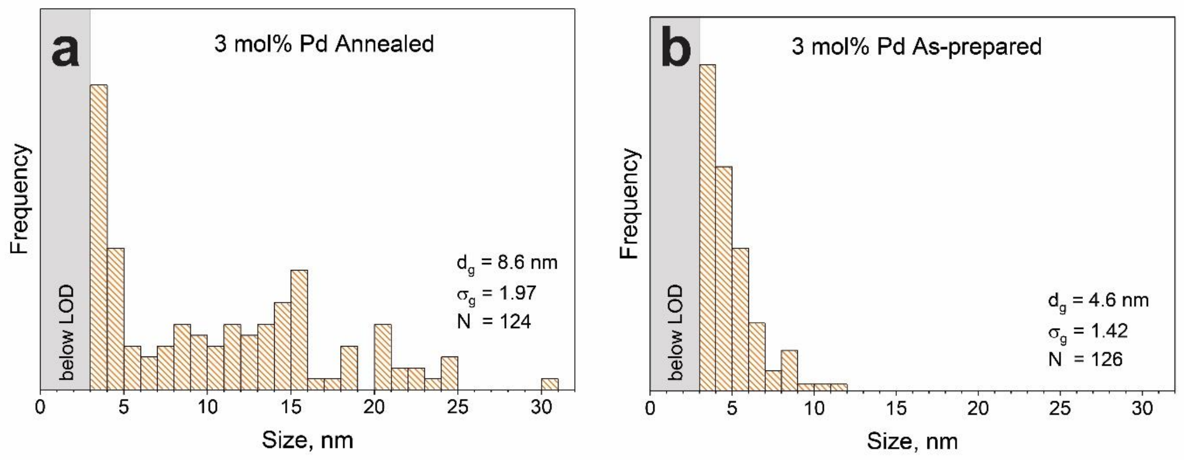

Figure S6: Area-equivalent Pd cluster size of 3 mol% Pd SnO_2 **a)** after and **b)** before annealing. Only clusters with diameters above 3 nm were considered, following the observations in Figure 2. The inset gives the geometric mean (d_g), geometric standard deviation (σ_g) and number of counted particles (N).

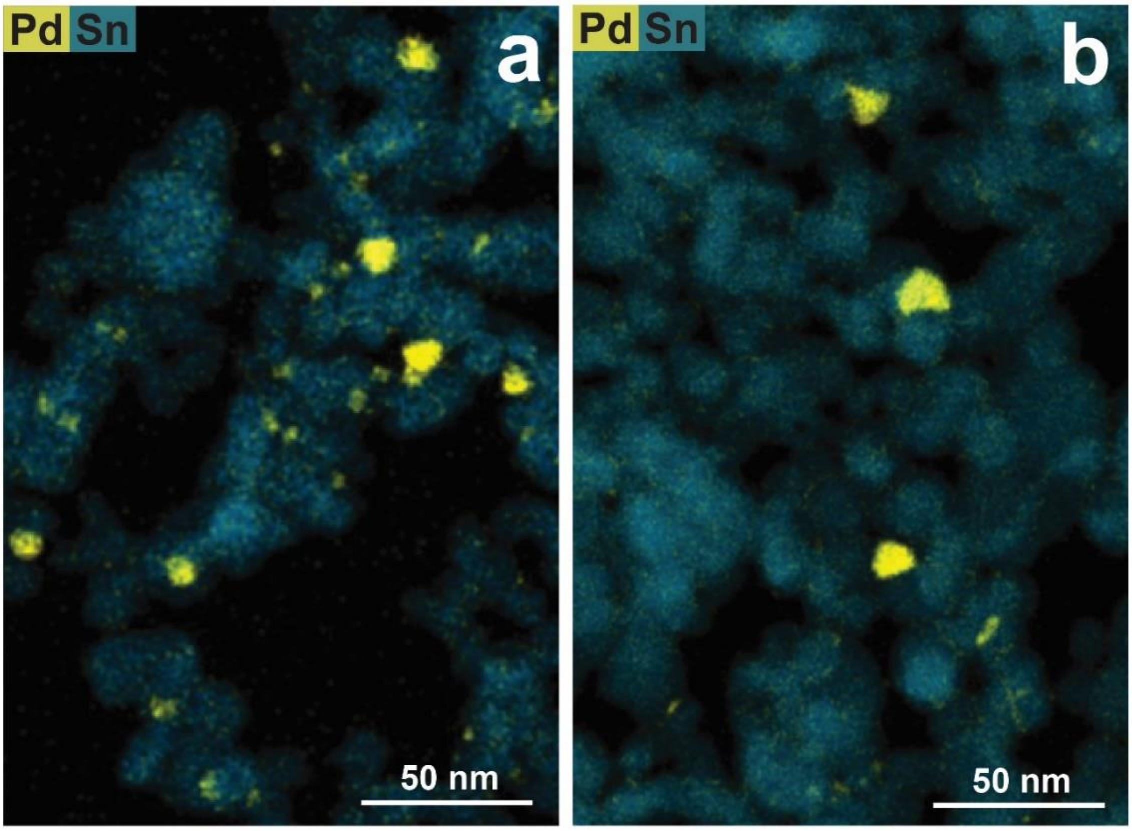

Figure S7: Additional microscopy images of 3 mol% Pd on SnO_2 with Pd and Sn elemental mappings of **a)** as-prepared and **b)** annealed after photodeposition. The as-prepared samples (a) show more Pd clusters, especially small ones (< 5 nm) that grow to larger ones (b).

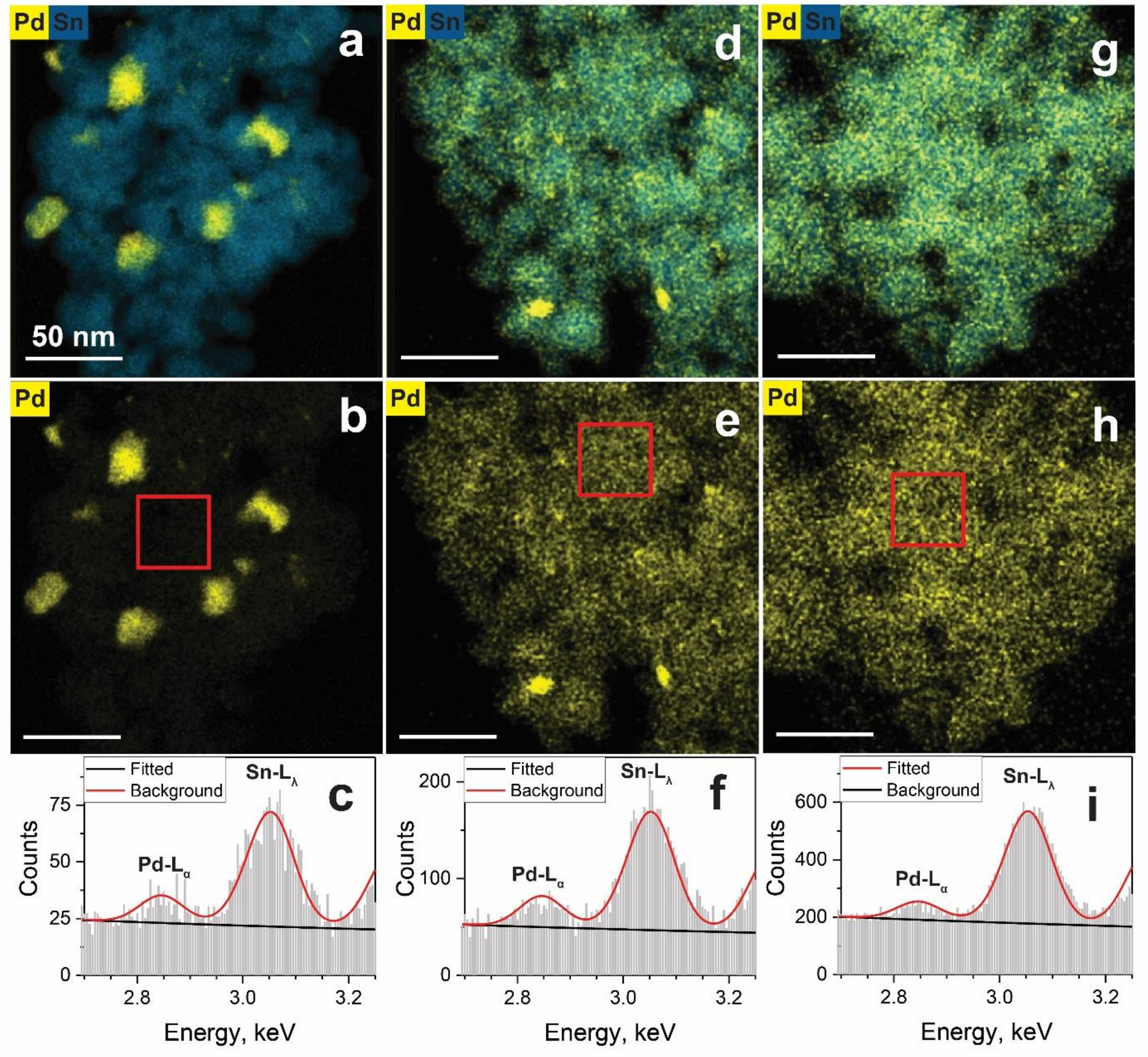

Figure S8: Elemental maps of annealed SnO_2 particles with **a-b)** 3 mol% Pd, **d-e)** 1 mol% Pd and **g-h)** 0.5 mol% Pd showing the overlay of Pd and Sn (**a,d,g**) or only Pd (**b,e,h**). All scale bars are 50 nm. For 3 mol% Pd, Pd particles from tens of nm down to a few nm can be observed. At 1 mol% Pd, Pd-particles with sizes between 15 and a few nm can be observed. At 0.5 mol%, barely any Pd-spots can be distinguished, indicating either very homogeneous distribution or spot sizes below 2 nm. Note that the color scale is adjusted for each image separately as the software of the microscope automatically adjusts the brightness to the brightest spot within the image. To allow a better comparison of the Pd loading in areas with weaker Pd signal (boxes in b,e,h), EDX spectra are shown in **c,f, and i**, revealing a very similar ratio between Pd and Sn, regardless of Pd loading.

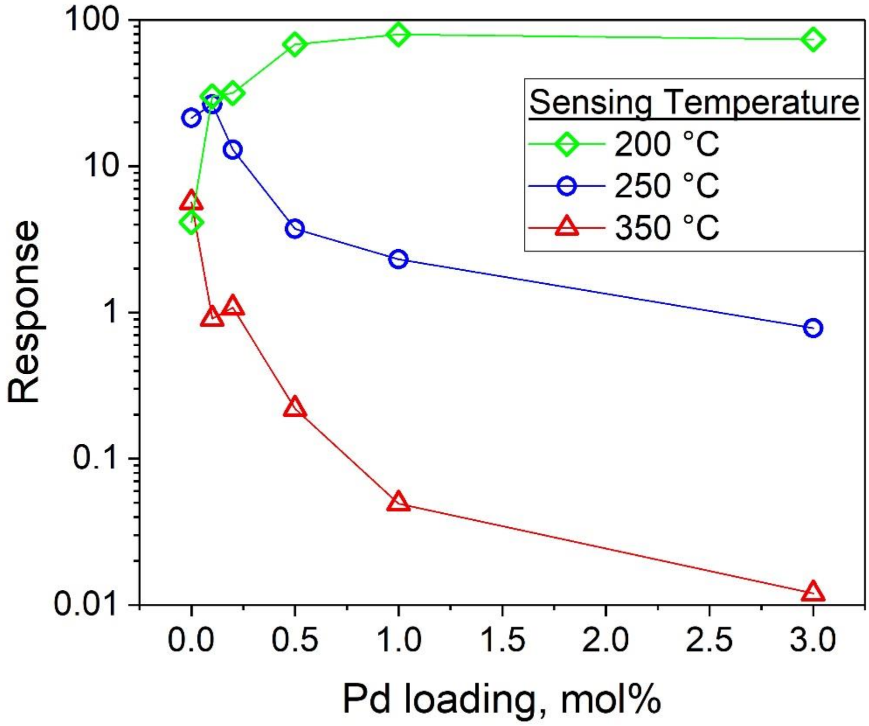

Figure S9: Responses to 1 ppm acetone (50 % RH) at different temperatures. Depending on the operating temperature, any Pd deteriorates sensing performance at 350 °C (red triangles). Only a tiny fraction of Pd improves sensing at 250 °C (blue circles), or any Pd loading improves sensing at 200 °C (green diamonds).

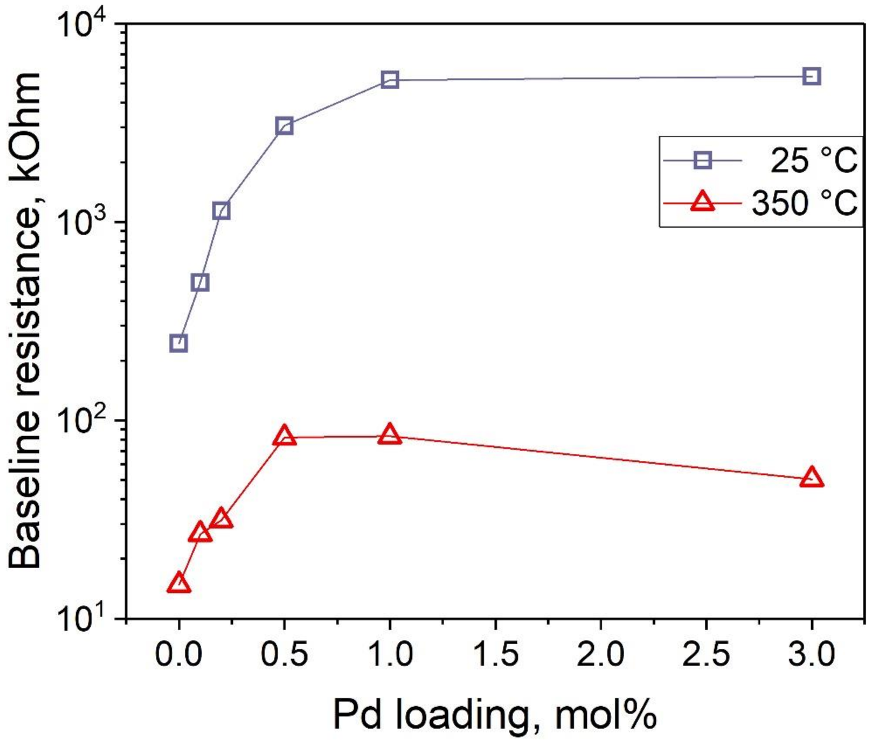

Figure S10: Baseline resistance (50 % relative humidity) at room temperature (purple squares) and 350 °C (red triangles). The increase of the resistance with Pd loading and saturation indicates the electrical sensitization effect of PdO_x on SnO_2 [25].

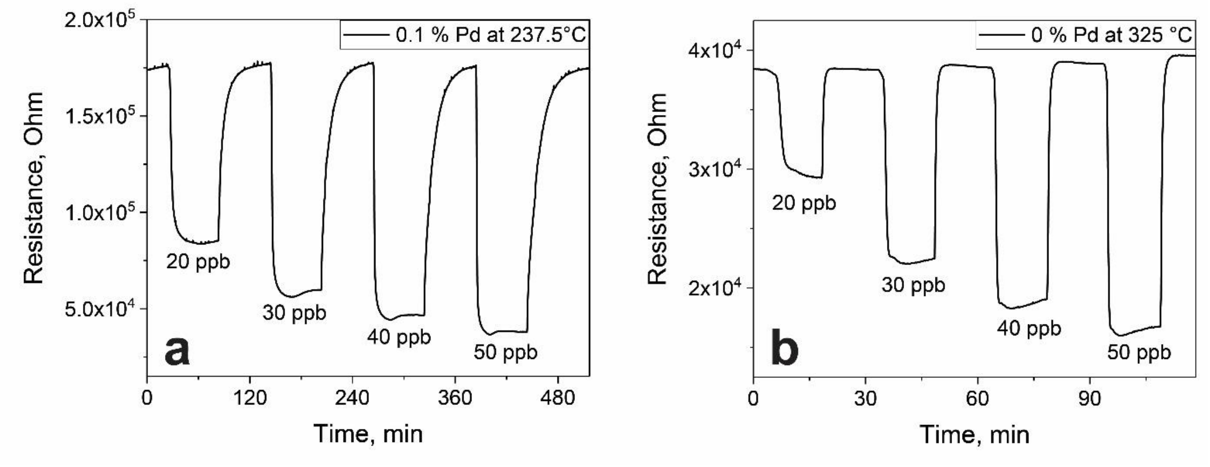

Figure S11: Film resistance in response to low concentration acetone pulses (20 – 50 ppb) of **a)** 0.1% Pd-SnO_2 operated at 237.5 °C and **b)** 0% Pd-SnO_2 operated at 325 °C and 50 % RH.

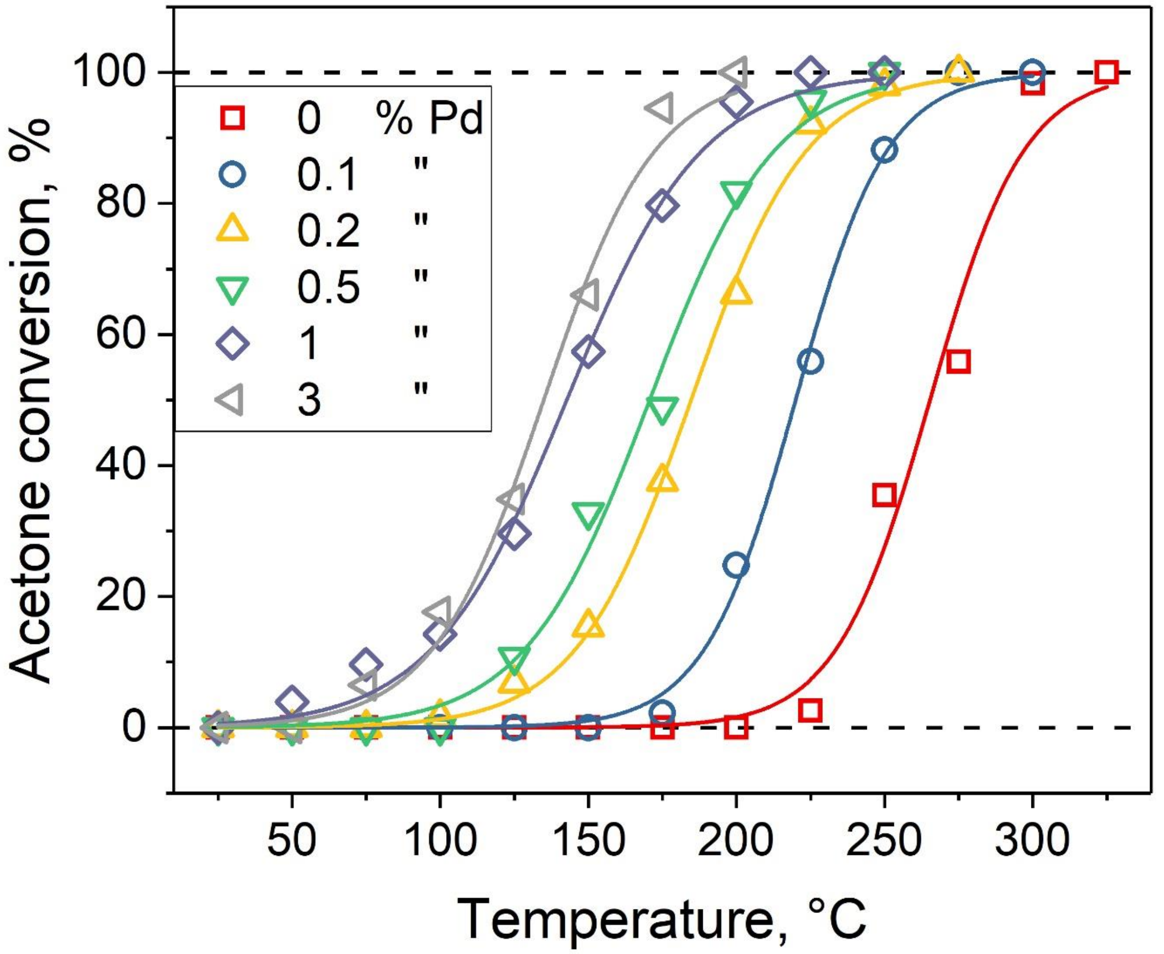

Figure S12: Acetone conversion fitting (solid lines) following Cabot et al. [9].

Table S1: Comparison of reported acetone sensors not based on Pd-SnO_2 with their key performance indicators

Material	Operating Temp. [°C]	Relative Humidity [%]	Response[a] (conc. In ppm)	Equiv. response at 1 ppm[b]	LOD [ppm]	Response time[c] [s]	Recovery time[c] [s]	Reference
Si-doped WO_3	400	90	1.48 (0.6)<br>Fig. 5a	2.47	0.02*<br>Fig. 7	77 (0.1 ppm)<br>Fig. 5b	83 (0.6 ppm)<br>Fig. 5b	Righettoni, et al., 2010
$ZnO/ZnCo_2O_4$	275<br>Fig. 7a	No R.H.	1.5 (10)<br>Fig. 8b	0.15	10*<br>Fig. 8b	15 (10 ppm)<br>Fig. 8b	38 (10 ppm)<br>Fig. 8b	Zhou, et al., 2014
Fe_2O_3	350<br>Fig. 4a	No R.H.	10.5 (1)<br>Fig. 4c	10.5	0.5*<br>0.0017†<br>text	3 (50 ppm)<br>Fig. 4b	120 (50 ppm)<br>Fig. 4b	Kim, et al., 2014
$ZnFe_2O_4$	200<br>Fig. 5a	No R.H.	0.4 (1)<br>Fig. 6a, inset	0.4	1*<br>Fig. 6a, inset	15 (20 ppm)<br>Fig. 5b	407 (20 ppm)<br>Fig. 5b	Zhou, et al., 2015
Au-modified In_2O_3	340<br>Fig. 5a	22	16 (1)<br>Fig. 7	16	0.02*<br>0.015†<br>Fig. 7	13<br>Fig. 6d	16<br>Fig. 6d	Xing, et al., 2015
Pt-doped SnO_2	400<br>Fig. 3a	90	5 (1.2)<br>Fig. 6a	4.2	0.25*<br>Fig. 6a	n.a.	n.a.	Güntner, et al., 2016
Pt-decorated In_2O_3	200<br>Fig. 7	No R.H.	10.4 (1.56)<br>Fig. 7	6.7	0.05*<br>0.01†<br>Fig. 8, inset	16 (1.56 ppm)<br>Fig. 6	67 (1.56 ppm)<br>Fig. 6	Karmaoui, et al., 2016
Rh-functionalized WO_3	350<br>Fig. 6a	95	11 (1)<br>Fig. 6b	11	1*<br>0.1†<br>Fig. 6c	20<br>Fig. 7b	92<br>Fig. 7b	Kim, et al., 2016
Pt-PS-SnO_2 NT	350<br>Fig. 5d	90	33.2 (1)<br>Fig. 5a	33.2	0.01*<br>0.01†<br>Fig. 5b	11 (5 ppm)<br>Fig. 5e	310 (5 ppm)<br>Fig. 5e	Jang, et al., 2016
Porous WO_3 NF	270<br>Fig. 8a	30 (55,75,95)	3.1 (1)<br>Fig. 8c	3.1	0.1*<br>Fig. 5c	12<br>Fig. 5c	13<br>Fig. 5c	Wei, et al., 2016
Yb-ferrites	230<br>Fig. 18	33	2.04 (1)<br>Fig. 18	2.04	0.05*<br>Fig. 18	29 (0.5 ppm)<br>Fig. 14b	55 (0.5 ppm)<br>Fig. 14b	Zhang, et al., 2017b
ZnO-Fe_3O_4	485	20	1.33 (1)	1.33	0.15*	16	19	

	Fig. 6		Fig. 8c		Fig. 8c	Fig. 7d	Fig. 7d	Zhang, et al., 2017a
PdO-Co_3O_4 HNC	350	90	0.55 (1)	0.55	0.4*<br>0.1†	194 (5 ppm)	350 (5 ppm)	Koo, et al., 2017
			Fig. 4a		Fig. 4a	Fig. 4d	Fig. 4d	
Pt-ZnO NFs	450	95	3.5 (1)	3.5	1*<br>0.029†	20	45	Cho, et al., 2017
			Fig. 5c		Fig. 5c	Fig. 6b	Fig. 6a	
PtRh-WO_3 NF	350	90	103 (1)	103	0.1*<br>0.0003†	4s	176	Kim, et al., 2017
			Fig. 2f		Fig. 2e	Fig. 3f	Fig. 3f	
Pt-Fe_2O_3	220	30	0.8 (1)	0.8	0.8*	1 (100 ppm)	46 (100 ppm)	Liu, et al., 2017
	Fig. 6a		Fig. 7c		Fig. 7c	Fig. 8a	Fig. 8a	
PtO_2-loaded SnO_2	400	95	21 (1)	21	0.4*	15	n.a.	Jeong, et al., 2018
			Fig. 3c		Fig. 3a	Fig. 3b		
Pt-decorated $CuFe_2O_4$	300	50	2.25 (5)	0.45	5*	15 (100 ppm)	360 (100 ppm)	Zhao, et al., 2018
	Fig. 5a		Fig. 5b		Fig. 5b	Fig. 6	Fig. 6	
La_2O_3-doped SnO_2	350	No R.H.	6 (1)	6	0.1*<br>0.08†	115	1068	Tammanoon, et al., 2018
	Fig. 9		Fig. 10c		Fig. 10c	Fig. 10c	Fig. 10b	
WO_3-SnO_2	360	No R.H.	11.1 (100)	0.11	100	11.5 (600 ppm)	7 (600 ppm)	Zhu, et al., 2018
	Fig. 3a		Fig. 3b		Fig. 3b	Fig. 3c	Fig. 3c	
$CuFe_2O_4$/Fe_2O_3	275	50	0.8 (1)	0.8	0.1*	5	60	Li, et al., 2018 [42]
	Fig. 6a		Fig. 7b		Fig. 7b	Fig. 8	Fig. 8	
Fe-C-WO_3	300	50 (also 20, 70, 90)	7 (1)	7	0.2*	19 (0.9 ppm)	21 (0.9 ppm)	Shen, et al., 2018
	Fig. 8		Fig. 9b		Fig. 9a	Fig. 11	Fig. 11	
Pt@HP WO_3 NFs	350	55 (also 20, 90)	11.2 (1)	11.2	0.4*	150	60	Kim, et al., 2018
	Fig. 5a		Fig. 5b		Fig. 5c	Fig. 5c	Fig. 5c	
WO_3/Pt-rGO	230	60 (also 0, 95)	1.1 (1)	1.1	1*<br>0.6†	14.1 (10 ppm)	16.8 (10 ppm)	Chen, et al., 2018
	Fig. 5		Fig. 7a		Fig. 7a	Fig. 6	Fig. 6	

Co_3O_4 core-shell	190<br>Fig. 4a	No R.H.	1.3 (10)<br>Fig. 4f, inset	0.13	10*<br>Fig. 4f, inset	4 (200 ppm)<br>Fig. 4c	8 (200 ppm)<br>Fig. 4c	Zhang, et al., 2018
Al-doped ZnO	450<br>Fig. 3d	90	12.5 (1)<br>Fig. 4c	12.5	0.1*<br>Fig. 4a	3 (10 ppm)<br>Fig. 3d	300 (10 ppm)<br>Fig. 3d	Yoo, et al., 2019
Au-containing ZnO	325<br>Fig. 3a	45	0.85 (1)<br>Fig. 3b, inset	0.85	0.2<br>Fig. 3b	6 (100 ppm)<br>Fig. 3d	3 (100 ppm)<br>Fig. 3d	Xu, et al., 2019
$W_{18}O_{49}/Ti_3C_2T_x$	300<br>Fig. 5f	10-98	1.5 (1)<br>Fig. 6a, inset	1.5	0.17*<br>Fig. 6a, inset	5.6 (20 ppm)<br>Fig. 5g	18.2 (20 ppm)<br>Fig. 5g	Sun, et al., 2020
$ZnCo_2O_4$@Ag	220<br>Fig. 5a	No R.H.	1.7 (1)<br>Fig. 5b, inset	1.7	0.25*<br>Fig. 5b, inset	6<br>Fig. 6d	25<br>Fig. 6d	Zhang, et al., 2020
$Bi_{1-x}La_xFeO_3$	260<br>Fig. 3c	90 (also 55, 70)	17 (1)<br>Fig. 4c	17	0.05*<br>Fig. 4a	15 (0.05 ppm)<br>Fig. 4e	13 (0.05 ppm)<br>Fig. 4e	Peng, et al., 2020
Au@ZnO NS	170<br>Fig. 9a	no	4.7 (1)<br>Fig. 10b, inset	4.7	0.5*<br>Fig. 10b, inset	27 (100 ppm)<br>Fig. 10c	18 (100 ppm)<br>Fig. 10c	Cao, et al., 2020
SnO_2	325	50	5.8 (1)	5.8	0.020*<br>$0.00005^{\dagger}$	24	104	This work
0.1 mol% Pd-loaded SnO_2	237.5	50	43.2 (1)	43.2	0.020*<br>$0.00005^{\dagger}$	26	1361	This work

a: Responses were converted to the definition used in this manuscript

b: If not available, response was linearly extrapolated

c: Concentration was 1 ppm if not stated otherwise

†: LOD calculated/estimated

*: lowest measured concentration

Table S2. Comparison of reported Pd-SnO_2 sensors for acetone detection with their key performance indicators. The precise origin of the literature is also given.

Material	Operating Temp. [°C]	Relative Humidity [%]	Response [a] (Conc. in ppm)	Equiv. Response at 1 ppm [b]	LOD [ppm]	Response Time [c] [s]	Recovery Time [c] [s]	Reference
Pd-SnO_2	300	Yes, but not specified	78 (25)	23.5	25 *<br>1 †	n.a.	n.a.	Epifani et al. (2008) [45]
	Fig. 8		Fig. 9		Fig. 9			
Pd-loaded flower-like SnO_2	250	No R.H.	10 (10)	1	10 *	11 (10 ppm)	30 (10 ppm)	Tian et al. (2014) [46]
	Fig. 9		Fig. 11		Fig. 11	Fig. 12	Fig. 12	
Pd-SnO_2 organized	RT	No R.H.	1.8 (10)	0.18	10 *	13	15	Shao et al. (2015) [34]
			Fig. 7c		Fig. 7c	Fig. 7b	Fig. 7b	
Pd-SnO_2 nanofibers	275	No R.H.	3 (1)	3	1 *	20	40	Tang et al. (2015) [33]
	Fig. 9		Fig. 10		Fig. 10	Fig. 11	Fig. 11	
Pd-loaded SnO_2 ultrathin nanorod-assembled hollow microspheres	230	No R.H.	10.6 (20)	0.5	n.a.	n.a.	n.a.	Zhang et al. (2017) [47]
			Fig. 8					
PdO@ZnO-SnO_2 NT	400	95	4.1 (1)	4.1	0.1 *<br>0.01 †	19.6	64	Koo et al. (2017) [48]
			Fig. 4b		Fig. 4a	Fig. 4c	Fig. 4d	
PdAu-SnO_2 nanosheets	250	40–70	2.7 (1)	2.7	0.1 *<br>0.045 †	5	4	Li et al. (2019) [49]
	Fig. 6a		Fig. 8a		Fig. 8a	Fig. 8b	Fig. 8b	
Pd-doped SnO_2	350	50	7 (1)	7	0.005 *<br>0.0005 †	60 (50 ppb)	138 (50 ppb)	Pineau et al. (2020) [18]
			Fig. 5a		Fig. 7	Fig. 7	Fig. 8	
SnO_2	325	50	5.8 (1)	5.8	0.020 *<br>0.00005 †	24	104	This work
0.1% Pd-loaded SnO_2	237.5	50	43.2 (1)	43.2	0.020 *<br>0.00005 †	26	1361	This work

a: Responses were converted to the definition used in this manuscript. b: If not available, response was linearly extrapolated. c: Concentration was 1 ppm if not stated otherwise. †: LOD calculated/estimated. *: Lowest measured concentration

Activation Energy Determination (Khan, et al., 2021)

Flow rate of acetone ($F_{Acetone}$):

$$F_{Acetone}\left[\frac{mol}{s}\right] = \frac{\dot{V}_{total} \cdot c_{Acetone}}{V^{M}_{Acetone}}$$

wherein $\dot{V}_{total}$, equals the total gas flow over the catalyst bed, $c_{Acetone}$ the acetone concentration and $V^{M}_{Acetone}$ the molar volume of acetone, which is 24.46 L/mol, assuming ideal gas at 25 °C.

Reaction rate:

$$\text{rate} = \frac{F_{Acetone} \cdot X_{Acetone}}{m_{Pd}}$$

wherein $X_{Acetone}$ equals the conversion of acetone, and m_{Pd}, the mass of Pd used.

Finally, the activation energy was calculated by taking the slope of an Arrhenius plot, represented by:

$$\ln(\text{rate}) = \frac{-E_A}{R} * \frac{1}{T}$$

wherein E_A is the activation energy (kJ/mol), R the ideal gas constant (J/(K*mol)), and T the absolute temperature (K).

As the temperature resolution of our catalytic experiments yielded insufficient amount of data points in the typically employed range (2-10% conversion) for these calculations, the catalytic conversion curves were fitted according to Cabot et al. [9] by

$$Conversion(T) = \frac{N_c}{N_c + N_{nc}} = \frac{1}{1+(N_{nc}/N_c)} \propto \frac{1}{1+e^{-(T-T_{50\%})/\beta}}$$

where N_c and N_{nc} are the number of converted and non-converted acetone molecules, respectively. The $T_{50\%}$ corresponds to the temperature of 50% of the catalytic conversion maximum, and β is the parameter giving the rate of variation of the catalytic conversion with temperature. The results of the fitting can be seen in Figure S12.

References

Cao, P. J.; Huang, Q. G.; Navale, S. T.; Fang, M.; Liu, X. K.; Zeng, Y. X.; Liu, W. J.; Stadler, F. J.; Lu, Y. M. *Appl. Surf. Sci.* **2020,** 518, 146223.

Chen, L.; Huang, L.; Lin, Y.; Sai, L.; Chang, Q.; Shi, W.; Chen, Q. *Sens. Actuators B Chem.* **2018,** 255, 1482-1490.

Cho, H.-J.; Kim, S.-J.; Choi, S.-J.; Jang, J.-S.; Kim, I.-D. *Sens. Actuators B Chem.* **2017,** 243, 166-175.

Fagherazzi, G.; Canton, P.; Riello, P.; Pernicone, N.; Pinna, F.; Battagliarin, M. *Langmuir* **2000,** 16, (10), 4539-4546.

Güntner, A. T.; Koren, V.; Chikkadi, K.; Righettoni, M.; Pratsinis, S. E. *ACS Sensors* **2016,** 1, (5), 528-535.

Jang, J.-S.; Choi, S.-J.; Kim, S.-J.; Hakim, M.; Kim, I.-D. *Adv. Funct. Mater.* **2016,** 26, (26), 4740-4748.

Jeong, Y. J.; Koo, W.-T.; Jang, J.-S.; Kim, D.-H.; Kim, M.-H.; Kim, I.-D. *ACS Appl Mater Inter* **2018,** 10, (2), 2016-2025.

Karmaoui, M.; Leonardi, S. G.; Latino, M.; Tobaldi, D. M.; Donato, N.; Pullar, R. C.; Seabra, M. P.; Labrincha, J. A.; Neri, G. *Sens. Actuators B Chem.* **2016,** 230, 697-705.

Khan, W. U.; Yu, I. K. M.; Sun, Y.; Polson, M. I. J.; Golovko, V.; Lam, F. L. Y.; Ogino, I.; Tsang, D. C. W.; Yip, A. C. K. *Environ. Pollut.* **2021,** 279, 116899.

Kim, D.-H.; Jang, J.-S.; Koo, W.-T.; Choi, S.-J.; Kim, S.-J.; Kim, I.-D. *Sens. Actuators B Chem.* **2018,** 259, 616-625.

Kim, D. H.; Shim, Y.-S.; Jeon, J.-M.; Jeong, H. Y.; Park, S. S.; Kim, Y.-W.; Kim, J.-S.; Lee, J.-H.; Jang, H. W. *ACS Appl Mater Inter* **2014,** 6, (17), 14779-14784.

Kim, N.-H.; Choi, S.-J.; Kim, S.-J.; Cho, H.-J.; Jang, J.-S.; Koo, W.-T.; Kim, M.; Kim, I.-D. *Sens. Actuators B Chem.* **2016,** 224, 185-192.

Kim, S.-J.; Choi, S.-J.; Jang, J.-S.; Cho, H.-J.; Koo, W.-T.; Tuller, H. L.; Kim, I.-D. *Adv. Mater.* **2017,** 29, (36), 1700737.

Koo, W.-T.; Yu, S.; Choi, S.-J.; Jang, J.-S.; Cheong, J. Y.; Kim, I.-D. *ACS Appl. Mater. Interfaces* **2017,** 9, (9), 8201-8210.

Liu, C.; Gao, H.; Wang, L.; Wang, T.; Yang, X.; Sun, P.; Gao, Y.; Liang, X.; Liu, F.; Song, H.; Lu, G. *Sens. Actuators B Chem.* **2017,** 252, 1153-1162.

Müller, M.; Mikoviny, T.; Feil, S.; Haidacher, S.; Hanel, G.; Hartungen, E.; Jordan, A.; Märk, L.; Mutschlechner, P.; Schottkowsky, R.; Sulzer, P.; Crawford, J. H.; Wisthaler, A. *Atmos. Meas. Tech.* **2014,** 7, (11), 3763-3772.

Peng, S.; Ma, M.; Yang, W.; Wang, Z.; Wang, Z.; Bi, J.; Wu, J. *Sens. Actuators B Chem.* **2020,** 313, 128060.

Righettoni, M.; Tricoli, A.; Pratsinis, S. E. *Anal. Chem.* **2010,** 82, (9), 3581-3587.

Shen, J.-Y.; Wang, M.-D.; Wang, Y.-F.; Hu, J.-Y.; Zhu, Y.; Zhang, Y. X.; Li, Z.-J.; Yao, H.-C. *Sens. Actuators B Chem.* **2018,** 256, 27-37.

Sun, S.; Wang, M.; Chang, X.; Jiang, Y.; Zhang, D.; Wang, D.; Zhang, Y.; Lei, Y. *Sens. Actuators B Chem.* **2020,** 304, 127274.

Tammanoon, N.; Wisitsoraat, A.; Phokharatkul, D.; Tuantranont, A.; Phanichphant, S.; Yordsri, V.; Liewhiran, C. *Sens. Actuators B Chem.* **2018,** 262, 245-262.

Wei, S.; Zhao, G.; Du, W.; Tian, Q. *Vacuum* **2016,** 124, 32-39.

Xing, R.; Li, Q.; Xia, L.; Song, J.; Xu, L.; Zhang, J.; Xie, Y.; Song, H. *Nanoscale* **2015,** 7, (30), 13051-13060.

Xu, S.; Zhang, H.; Qi, L.; Xiao, L. *Microchim Acta* **2019,** 186, (6), 342.

Yoo, R.; Güntner, A. T.; Park, Y.; Rim, H. J.; Lee, H.-S.; Lee, W. *Sens. Actuators B Chem.* **2019,** 283, 107-115.

Zhang, D.; Jin, Y.; Chen, H.; Luo, Y.; Zhang, Y. *Ceram. Int.* **2020,** 46, (10), 15176-15182.

Zhang, L.; Dong, B.; Xu, L.; Zhang, X.; Chen, J.; Sun, X.; Xu, H.; Zhang, T.; Bai, X.; Zhang, S.; Song, H. *Sens. Actuators B Chem.* **2017a,** 252, 367-374.

Zhang, P.; Qin, H.; Lv, W.; Zhang, H.; Hu, J. *Sens. Actuators B Chem.* **2017b,** 246, 9-19.

Zhang, R.; Zhou, T.; Wang, L.; Zhang, T. *ACS Appl Mater Inter* **2018,** 10, (11), 9765-9773.

Zhao, C.; Lan, W.; Gong, H.; Bai, J.; Ramachandran, R.; Liu, S.; Wang, F. *Ceram. Int.* **2018,** 44, (3), 2856-2863.
Zhou, X.; Feng, W.; Wang, C.; Hu, X.; Li, X.; Sun, P.; Shimanoe, K.; Yamazoe, N.; Lu, G. *J. Mater. Chem. A* **2014,** 2, (41), 17683-17690.
Zhou, X.; Li, X.; Sun, H.; Sun, P.; Liang, X.; Liu, F.; Hu, X.; Lu, G. *ACS Appl Mater Inter* **2015,** 7, (28), 15414-15421.
Zhu, L.; Zeng, W.; Li, Y. *Mater. Lett.* **2018,** 231, 5-7.
